# Polymorphisms in the Mitochondrial Ribosome Recycling Factor *EF-G2mt/MEF2* Compromise Cell Respiratory Function and Increase Atorvastatin Toxicity

**DOI:** 10.1371/journal.pgen.1002755

**Published:** 2012-06-14

**Authors:** Sylvie Callegari, Philip A. Gregory, Matthew J. Sykes, Jennifer Bellon, Stuart Andrews, Ross A. McKinnon, Miguel A. de Barros Lopes

**Affiliations:** 1 School of Pharmacy and Medical Sciences, Division of Health Sciences, University of South Australia, Adelaide, South Australia, Australia; 2 Division of Human Immunology, Centre for Cancer Biology, SA Pathology, Adelaide, South Australia, Australia; 3 Discipline of Medicine, The University of Adelaide, Adelaide, South Australia, Australia; 4 Sansom Institute, Division of Health Sciences, University of South Australia, Adelaide, South Australia, Australia; 5 Australian Wine Research Institute, Glen Osmond, South Australia, Australia; 6 Flinders Centre for Innovation in Cancer, School of Medicine, Flinders University, Bedford Park, South Australia, Australia; University of Tokyo, Japan

## Abstract

Mitochondrial translation, essential for synthesis of the electron transport chain complexes in the mitochondria, is governed by nuclear encoded genes. Polymorphisms within these genes are increasingly being implicated in disease and may also trigger adverse drug reactions. Statins, a class of HMG-CoA reductase inhibitors used to treat hypercholesterolemia, are among the most widely prescribed drugs in the world. However, a significant proportion of users suffer side effects of varying severity that commonly affect skeletal muscle. The mitochondria are one of the molecular targets of statins, and these drugs have been known to uncover otherwise silent mitochondrial mutations. Based on yeast genetic studies, we identify the mitochondrial translation factor *MEF2* as a mediator of atorvastatin toxicity. The human ortholog of *MEF2* is the Elongation Factor Gene (EF-G) 2, which has previously been shown to play a specific role in mitochondrial ribosome recycling. Using small interfering RNA (siRNA) silencing of expression in human cell lines, we demonstrate that the *EF-G2mt* gene is required for cell growth on galactose medium, signifying an essential role for this gene in aerobic respiration. Furthermore, *EF-G2mt* silenced cell lines have increased susceptibility to cell death in the presence of atorvastatin. Using yeast as a model, conserved amino acid variants, which arise from non-synonymous single nucleotide polymorphisms (SNPs) in the *EF-G2mt* gene, were generated in the yeast *MEF2* gene. Although these mutations do not produce an obvious growth phenotype, three mutations reveal an atorvastatin-sensitive phenotype and further analysis uncovers a decreased respiratory capacity. These findings constitute the first reported phenotype associated with SNPs in the *EF-G2mt* gene and implicate the human *EF-G2mt* gene as a pharmacogenetic candidate gene for statin toxicity in humans.

## Author Summary

The mitochondria are responsible for producing the cell's energy. Energy production is the result of carefully orchestrated interactions between proteins encoded by the mitochondrial DNA and by nuclear DNA. Sequence variations in genes encoding these proteins have been shown to cause disease and adverse drug reactions in patients. The cholesterol-lowering drugs statins are one class of drugs that interfere with mitochondrial function. Statins are one of the most prescribed drugs in the western world, but many users suffer side effects, commonly muscle pain. In severe cases this can lead to muscle breakdown and liver failure. In this study, we discover that disruption of a mitochondrial translation gene, *EF-G2mt*, impedes respiration and increases cell death when exposed to statin. Using the simple unicellular organism yeast as a model, the activity of naturally occurring human *EF-G2mt* variants is tested. Three of these variants render yeast cells more sensitive to statin. Patients who possess these *EF-G2mt* variations may be more susceptible to statin side effects. Importantly, the test for statin sensitivity also led to the discovery of mutants that have a reduced energy production capacity. The decreased ability to produce energy is linked to a number of diseases, including myopathies and liver failure.

## Introduction

The primary function of the mitochondria is the aerobic production of ATP, a process that is reliant on a series of protein complexes that comprise the electron transport chain. Several components of the electron transport chain are encoded in the mitochondrial genome, the translation of which is governed largely by nuclear encoded genes. Increasingly, mutations within these genes are being implicated with respiratory deficiency, an underlying factor in a number of diseases, including myopathies and liver failure [1], [2], [3], [4]. For example, pathogenic mutations in the human mitochondrial elongation factor genes, *EF-G1mt* and *EF-Tu(mt)*, have been implicated with severe lactic acidosis and encephalopathy [1], [2], [3]. Recently a mutation in a novel gene, believed to be a member of the class of mitochondrial peptide release factors, was identified in patients exhibiting symptoms of Leigh syndrome [4].

In addition to disease, there is also emerging evidence that respiratory deficiencies are responsible for adverse drug reactions. Consequently, treatment with certain drugs have uncovered otherwise silent mitochondrial mutations [5], [6]. The group of cholesterol-lowering drugs, statins, are one example. The primary target of statins is 3-hydroxy-3-methylglutaryl-coenzyme A (HMG-CoA) reductase, the rate limiting enzyme of the sterol synthesis pathway, but increasingly, studies are reporting signs of statin-induced mitochondrial dysfunction [7], [8]. This is believed to be a factor in the myopathic side-effects of statins. Approximately 0.1 to 0.5 percent of statin users experience severe myopathic symptoms (defined as serum creatine kinase levels more than 10 times the upper limit of normal) and many more suffer milder musculoskeletal pain [9], [10]. Frequently such patients present symptoms that are similar to those of patients with mitochondrial myopathies [11]. To date, there have been several case studies reporting the presence of a subclinical MELAS (mitochondrial encephalopathy, lactic acidosis and stroke-like episodes) mutation within the mitochondrial DNA (mtDNA) of patients who have developed severe myopathic symptoms following statin medication [12], [13], [14]. It is expected that existing weakness in mitochondrial function can be exacerbated upon exposure to statin, leading to the uncovering of previously asymptomatic mutations in mitochondrial genes.

The yeast *Saccharomyces cerevisiae* has been the model of choice for studies of mitochondrial function. In addition to mitochondrial similarities with human cells, the ability of yeast to survive in the absence of mtDNA, the simplicity with which both nuclear and mtDNA can be manipulated and the extensive number of tools and resources available specifically for yeast research has greatly contributed to an understanding of potentially pathogenic mutations [15], [16], [17]. Statins were first isolated as secondary metabolites from fungi, the presumption being that the strong antifungal properties of statins provide an ecological advantage for the producer over other fungi, similar to that of antibiotics. We and others have demonstrated that upon exposure to statin, yeast, as well as having reduced cell viability, also display evidence of mitochondrial dysfunction [18], [19], [20].

In this study, we identify a nuclear gene encoding a mitochondrial translation factor as a modulator of atorvastatin toxicity in yeast (*MEF2*) and human cell lines (EF-*G2mt*). The eukaryotic mitochondrial protein synthesis system consists of four phases; initiation, elongation, termination and ribosome recycling, each carefully orchestrated by a series of nuclear encoded proteins [21], [22]. The human *EF-G2mt* gene, originally named a mitochondrial elongation factor based on sequence homology with bacterial EF-G, has since been shown to function as a ribosome recycling factor [23], [24]. EF-G2mt is believed to interact with the already known ribosome recycling factor (RRF1) to promote dissociation of the ribosomal subunits following termination of translation [23]. In bacteria, the dual role of translocation and ribosome recycling are shared by a single EF-G protein [25]. Eukaryotic cells harbour two EF-G proteins in their mitochondria and it appears that these have distinct functions, the EF-G1mt protein for translocation and the EF-G2mt protein for ribosome recycling [23], [26]. The human EF-G2mt protein is conserved across the majority of eukaryotic species [23]. With its yeast Mef2p counterpart, the human EF-G2mt protein shares greater than 32 percent homology and four of the five protein domains. We use the atorvastatin-sensitive phenotype of the yeast *MEF2* gene to uncover naturally occurring human variants of EF-G2mt that have respiratory deficient phenotypes. These findings have ramifications for patient drug response and possibly also for disease.

## Results

### Identification of the yeast *MEF2* gene as a cell mediator of atorvastatin toxicity

In light of the emerging evidence that mitochondria are important in dictating statin toxicity, which in turn can reveal underlying respiratory defects that have important health implications, experiments were designed to discover mitochondrial lesions that affect statin sensitivity. Two published fitness profiling experiments in yeast have observed statin sensitivity in hundreds of heterozygous deletion mutants following statin exposure for 20 generations of growth [27], [28]. Using the Gene Ontology term finder available on the *Saccharomyces* genome database website (www.yeastgenome.org), we discovered approximately 14–17% of genes conferring statin-sensitivity are associated with the mitochondria. One of the most sensitive of these mitochondrial associated genes was the *MEF2* gene, encoding a mitochondrial translation factor believed to have a role in ribosome recycling [23], [27].

**Figure 1:**
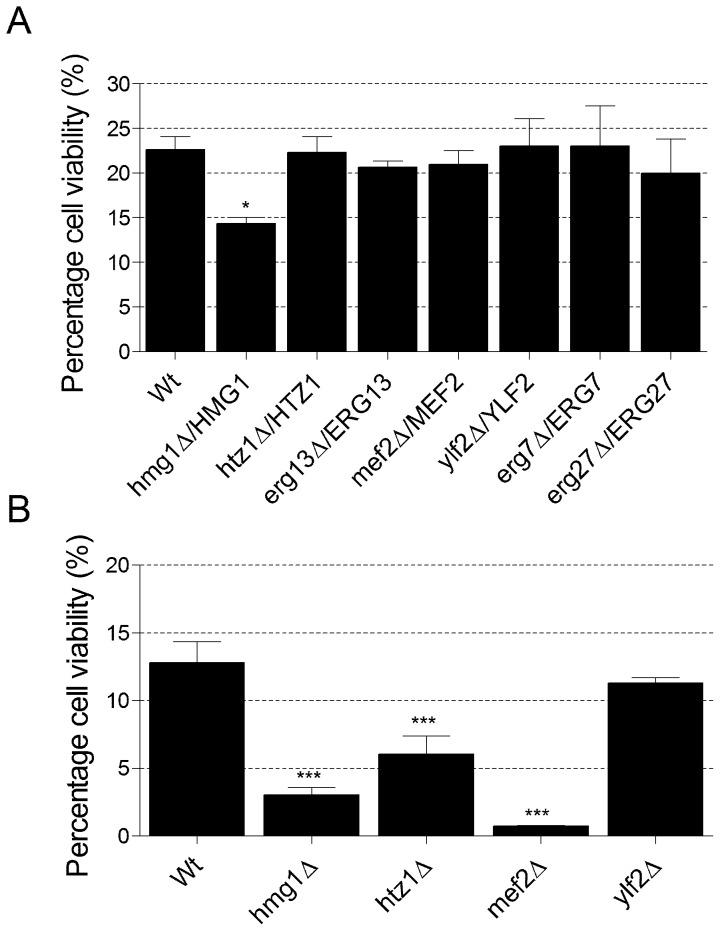
Viability of yeast heterozygous and haploid deletion mutants in atorvastatin after 5 days. (A) Percentage cell viability of heterozygous deletion mutants in 110 µM atorvastatin relative to viability in the solvent control. (B) Percentage cell viability of haploid deletion mutants in 110 µM atorvastatin relative to viability in the solvent control. Data represent mean ± SEM (n = 3). A one-way ANOVA, followed by a Dunnett's multiple comparison test, was used to compare the mean percentage viability of the mutant strains to that of the wild-type. *P<0.05, ***P<0.001.

The growth of deletion mutants in a competition style assay is a very sensitive method of detecting differences in growth rate. However, these assays are prone to a higher incidence of false positive and non-replicable results [29]. To confirm the *MEF2* phenotype, a number of yeast deletion mutants that ranked as the most statin sensitive in the fitness profiling experiments were compared [27], [28]. Both heterozygous and haploid mutants were tested and cell viability was assessed after five days exposure to 110 µM atorvastatin. The concentration of 110 µM atorvastatin is approximate to those concentrations used in the original genome-wide fitness profiling screens (62.5 µM and 125 µM atorvastatin) [27]. Furthermore, during a previous investigation of the effects of different atorvastatin concentrations in yeast, we have shown that 110 µM atorvastatin does not inhibit cell growth but is sufficient to cause a significant decrease in intracellular ergosterol (approximately 85%), accompanied by loss of cell viability after prolonged exposure (5 days) [18]. Of the heterozygous mutants tested, only the *hmg1*Δ/*HMG 1* strain was confirmed to be sensitive to atorvastatin (Figure 1A). However, of the haploid mutants tested, three displayed a statin hypersensitive phenotype (Figure 1B). The *mef2*Δ mutant emerged as the strain which exhibited the greatest loss of cell viability in the presence of atorvastatin, with an almost 20-fold reduction in cell viability compared with the wild-type. The *hmg1*Δ strain displayed a 5-fold reduction in cell viability and disruption of the *HTZ1* gene, encoding a histone protein, resulted in a 2-fold loss of cell viability (Figure 1B).

Yeast mutants defective in mitochondrial translation undergo rapid loss of mtDNA and we have previously shown this to be the case for the *mef2*Δ mutant [30]. Consequently, *mef2*Δ is ρ^0^ (completely devoid of mtDNA). To determine whether atorvastatin-sensitivity is the consequence of abrogation of the *MEF2* gene or simply from the absence of mtDNA, *mef2*Δ statin sensitivity was compared with that of ethidium bromide generated cytoplasmic ρ^0^ mutants. In order to ensure that there were no secondary site mutations in the *mef2*Δ deletion mutant that originated from the *S. cerevisiae* gene deletion collection, new *mef2*Δ haploid strains were created. Results show that although the ρ^0^ strains were more sensitive to atorvastatin than the respiratory positive parent, they did not display the same degree of sensitivity as *mef2*Δ (Table 1). Therefore, mutation of the *MEF2* gene is a critical determinant of statin sensitivity through mtDNA dependent and independent functions.

**Table 1:** Percentage cell viability relative to solvent control after 5 days exposure to 110 µM atorvastatin.

	Mean % viability ± SEM^a^
Wild-type	18.2±2.66***
*mef2*Δ	1.02±0.194
ρ^0^	8.01±0.720**

a Three independent replicates were performed.

A one-way ANOVA, followed by a Dunnett's multiple comparison test, was used to compare the viability of the *mef2*Δ strains to that of the wild-type and ρ^0^ strains.

**: *P*<0.01,

***: *P*<0.001.

### Silencing of the human *EF-G2mt* gene compromises OXPHOS and exacerbates atorvastatin toxicity in human cell lines

The human *EF-G2mt* gene, ortholog of the yeast *MEF2* gene, encodes a recently characterised mitochondrial ribosome recycling factor [23], but to date, no functional analysis has been performed for this gene. To determine whether the *EF-G2mt* gene is essential for human cell function and to ascertain whether depletion influences statin toxicity, an siRNA pool comprising of four individual *EF-G2mt* targeted siRNAs was used to silence *EF-G2mt* expression in the human rhabdomyosarcoma (RD) cell line. The RD cell line has previously been established as a skeletal muscle model for mitochondrial disorders and has also been used in studies of statin toxicity [31], [32], [33]. At 72 hours post-transfection, greater than 80 percent silencing was consistently achieved and cells remained viable. Cells were then re-transfected at this time point to enable continued depletion of *EF-G2mt* activity. This strategy had previously uncovered an essential role for cell viability for the first discovered ribosomal recycling factor gene, *RRF1* [34]. However, at 72 hours post-re-transfection (six days after the initial transfection), RD cells remained viable even though *EF-G2mt* mRNA concentration had decreased by 99.9 percent. Additionally, there was no decrease in mtDNA levels upon *EF-G2mt* silencing as analysed using quantitative PCR (Figure S1) [35].

**Figure 2:**
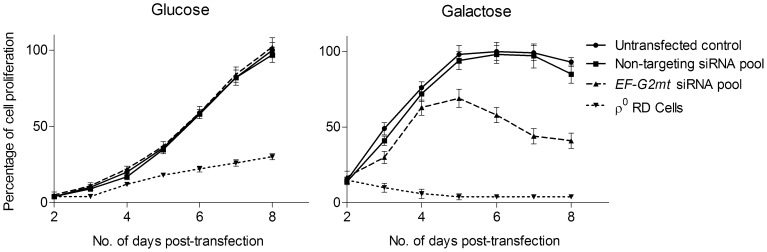
Growth of siRNA transfected RD cells in glucose and galactose medium. At 24 hours post-transfection, cells were seeded into wells of a 96-well plate in DMEM medium containing 10% fetal bovine serum and either 4.5 g/L glucose (left panel) or 4.5 g/L galactose (right panel). Cell proliferation was determined daily using a luminescent cell viability assay. An untransfected cell line and the rho0 cell line were included as controls. Cell proliferation is shown as a percentage of the maximum cell growth (100%) of the untransfected control. Error bars represent the mean ± SEM for three independent measurements.

Although gross inhibition of mitochondrial translation in human cell lines results in loss of cell viability [34], a more subtle mitochondrial phenotype may be masked by the phenomenon of the Crabtree effect whereby many human cell lines, when grown in the presence of glucose, derive their energy almost solely by fermentative means [36]. To circumvent this effect and force cells to rely on mitochondrial respiration as their primary energy source, galactose was used to replace glucose as the carbon source [37]. Silencing of *EF-G2mt* led to a marked decline in the growth of RD cells at four days post-transfection, which was maintained for a period of seven days (Figure 2). This growth defect on galactose medium signals an impairment in oxidative phosphorylation (OXPHOS).

Based on the findings in yeast, it was predicted that decreased *EF-G2mt* activity would also enhance the effects of atorvastatin toxicity. *EF-G2mt* silenced RD cells were subjected to various concentrations of atorvastatin in medium containing either galactose or glucose as the carbon source for a period of 48 hours. In glucose medium, there was no difference in statin sensitivity between the *EF-G2mt* silenced cells and those transfected with the non-targeting control. However, based on IC_50_ values (defined here as a 50% loss of viability at 48 hours) in galactose medium, *EF-G2mt* silenced cells were over 20 percent more sensitive to atorvastatin than cells transfected with the non-targeting siRNA pool (Table 2). These results confirm a role for the human *EF-G2mt* gene in cell resistance to atorvastatin in a human skeletal muscle cell line. Notably, a similar increase in sensitivity (17%) was observed using the human hepatic HepG2 cell line (a model for statin-induced liver toxicity), although it should be noted that HepG2 cells are approximately 10 times more statin resistant than RD cells and this elevation in atorvastatin sensitivity was not statistically significant in these experiments (Table 2).

**Table 2:** IC_50_ values for *EF-G2mt* silenced cells exposed to atorvastatin.

		IC_50_	95% confidence interval	*P*
**RD Cells**	Untransfected control	18.9	16.1 to 22.2	
	Non-targeting siRNA	18.6	15.7 to 22.0	
	*EF-G2mt* targeting siRNA	15.0*	13.8 to 16.4	0.023*
**HepG2 Cells**	Untransfected control	186.9	149.1 to 234.3	
	Non-targeting siRNA	184.8	148.9 to 228.8	
	*EF-G2mt* targeting siRNA	152.4	129.3 to 179.6	0.138

IC50 values of *EF-G2mt* silenced cells were compared to their respective non-targeting siRNA transfected cell lines using the extra sum-of-squares F-test.

*: *P*<0.05.

### EF-G2mt protein variants, when created in yeast Mef2p, increase atorvastatin toxicity

**Figure 3:**
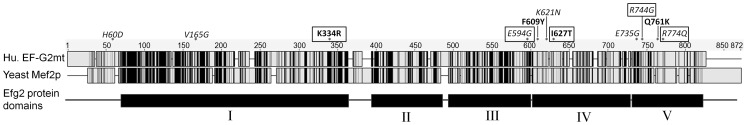
EF-G2mt protein variants. Alignment of the protein amino acid sequence of the human EF-G2mt protein with the yeast Mef2 protein. Dark shaded areas represent conserved amino acid residues and grey shaded areas represent semi-conserved residues. *EF-G2mt* SNPs that are semiconserved in yeast *MEF2* are shown in italics and fully conserved SNPs are depicted in bold. The five alleles selected for functional characterisation are outlined. The five EF-G2mt protein domains are represented below the alignment. Global alignment of protein sequences was performed using Lalign and the BioEdit sequence alignment editor was used to generate the graphical representation.

A global alignment of the amino acid sequence of the human EF-G2mt protein (Isoform I, AAH15712.1) with the yeast Mef2 protein (CAA59392) reveals 32.1% amino acid sequence identity (Figure 3 and Figure S2). At the commencement of this study, there were nine published non-synonymous Single Nucleotide Polymorphisms (SNPs) in the human *EF-G2mt* gene, of which five were either conserved or semi-conserved in the yeast *MEF2* gene. Three of these variants, *EF-G2mt* ^I627T^, *EF-G2mt* ^E594G^ and *EF-G2mt* ^K334R^, are considered rare, with a heterozygosity frequency below one percent. One of the variants, *EF-G2mt* ^R744G^, has a heterozygosity frequency of three percent and the *EF-G2mt* ^R774Q^ allele has a heterozygosity frequency greater than 20 percent. These five SNPs were selected for functional analysis using the yeast *MEF2* gene as a model.

**Figure 4:**
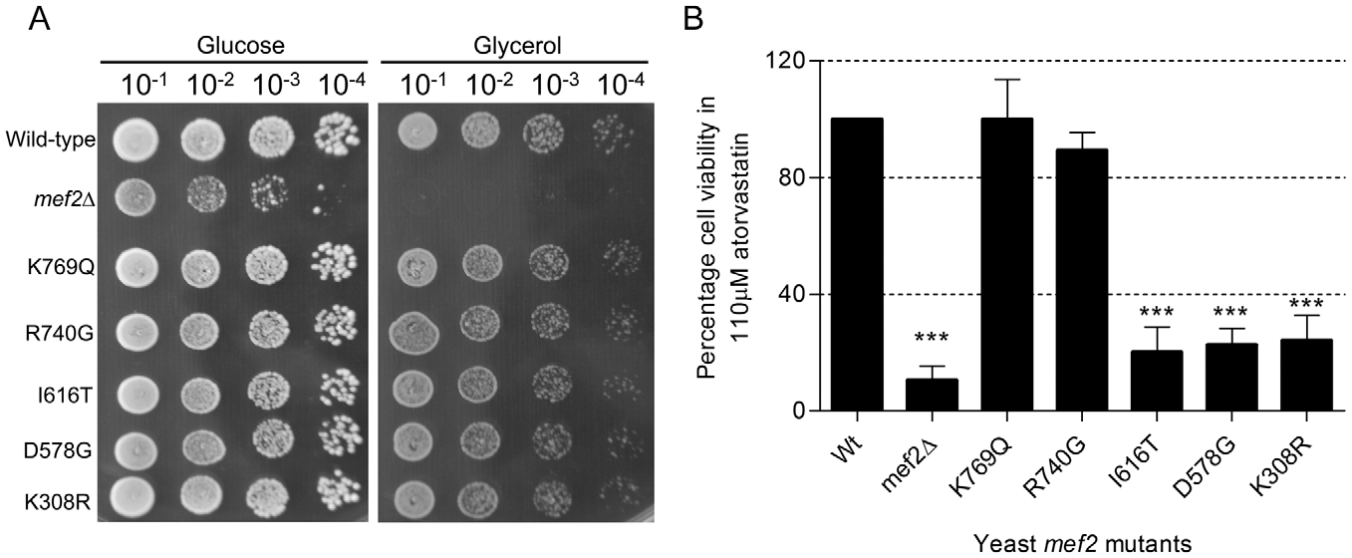
Functional characterisation of *EF-G2mt* equivalent SNPs in the yeast *MEF2* gene. (A) Growth of wild-type, *mef2* deletion strain and *mef2* mutants in yeast medium containing either glucose as the carbon source or the non-fermentable carbon source glycerol. Cells from exponentially growing cultures were serially diluted and 5 µl of each dilution spotted onto each plate. Growth was assessed after 72 hours incubation at 30°C. (B) Percentage cell viability of yeast *mef2* mutants in 110 µM atorvastatin relative to viability of the wild-type strain following exposure to atorvastatin for 5 days. Data represent mean ± SEM (n = 3). A one-way ANOVA followed by a Dunnett's multiple comparison test was used to compare the mean percentage viability of the *mef2* variants to that of the wild-type. ***P<0.001.

For each of the five selected human EF-G2mt variants, single nucleotide base pair substitutions were constructed directly into the chromosomal copy of the yeast *MEF2* gene to replace the codon specific to the wild-type amino acid residue with a codon that corresponds to the amino acid present in the human EF-G2mt protein variants. The Mef2p variants constructed were *mef2* ^K769Q^, *mef2* ^R740G^, *mef2* ^I616T^, *mef2* ^D578G^ and *mef2* ^K308R^ which correspond to *EF-G2mt* ^R774Q^, *EF-G2mt* ^R744G^, *EF-G2mt* ^I627T^, *EF-G2mt* ^E594G^ and *EF-G2mt* ^K334R^ respectively. To assess for respiratory competence, *mef2* mutants were grown on medium containing the non-fermentable carbon source glycerol. After 72 hours, all five mutants were proficient in the production of colonies on both glucose and glycerol medium. The number and size of colonies produced by the mutant strains on glycerol medium was equal to that of the wild-type, indicating that all mutants are respiratory competent (Figure 4A). Moreover, based on measurements of cell growth in both glucose and glycerol liquid medium, there were no growth defects exhibited by any of the *mef2* mutants (Table S1). Mitochondrial DNA stability was measured periodically for up to 32 generations. The frequency of cells which spontaneously lose mtDNA amongst populations of each *mef2* mutant remained equal to that of the wild-type (approximately 2 to 3%), verifying that mtDNA is stable over successive generations.

The five *mef2* mutants were then assayed for atorvastatin sensitivity. Following five days of exposure to 110 µM atorvastatin, three of the *mef2* mutants exhibited a statin hypersensitive phenotype. Viability of the *mef2* ^I616T^, *mef2* ^D578G^ and *mef2* ^K308R^ mutants was reduced to 20.3, 22.8 and 24.2 percent respectively (Figure 4B). The two other *mef2* mutants, *mef2* ^K769Q^ and *mef2* ^R740G^, did not exhibit a statin sensitive phenotype. The unmasking of a phenotype for the *mef2* ^K308R^, *mef2* ^D578G^ and *mef2* ^I616T^ variants by atorvastatin is a strong indicator that these mutations have an effect on Mef2p function. A similar effect conferred by these alleles in the human EF-G2mt protein could have vital consequences for statin users.

### Atorvastatin-sensitive *mef2* mutants also have reduced respiration rates

Although no obvious growth phenotype was observed, the statin-sensitive phenotype of three of these mutants indicates a subtle defect in mitochondrial function. Staining of *mef2* mutant cells with the nucleic acid staining dye 4′,6-diamidino-2-phenylindol (DAPI) confirmed the presence of mtDNA nucleoids in cells of all five mutants and quantification of mtDNA copy number using quantitative PCR (qPCR) [38] showed that mtDNA levels were the same as that of the wild-type (Figure S3). It therefore appears that the EF-G2mt equivalent mutations do not destabilise Mef2p function so as to compromise mtDNA stability.

**Figure 5:**
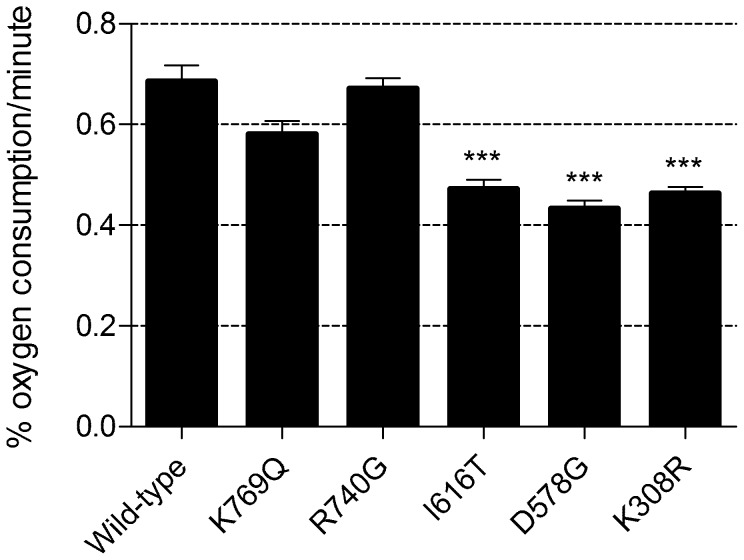
Oxygen consumption rates of *mef2* mutant cultures. Figure depicts the rate of oxygen consumption per minute per 30 mL of yeast culture at an OD_600_ of 0.2. Data represent mean ± SEM (n = 3). A one-way ANOVA followed by a Dunnett's multiple comparison test was used to compare the mean oxygen consumption rate of *mef2* variants to that of the wild-type. ***P<0.001.

To investigate the possibility of a respiratory phenotype, oxygen consumption for the three statin-sensitive *mef2* mutant cultures was measured (in the absence of atorvastatin) using a non-invasive oxoluminescent device [35]. All three mutants, *mef2* ^K308R^, *mef2* ^D578G^ and *mef2* ^I616T^, exhibited a significantly reduced respiration capacity, approximately one third lower than that of the wild-type (Figure 5). The two *mef2* mutants that did not display an atorvastatin-sensitive phenotype had respiration rates much closer to that of the wild-type. Together, these results demonstrate a respiratory phenotype conferred by at least three of the *EF-G2mt* equivalent *mef2* variants and this correlates with the inability of these *mef2* mutants to tolerate atorvastatin toxicity.

**Figure 6:**
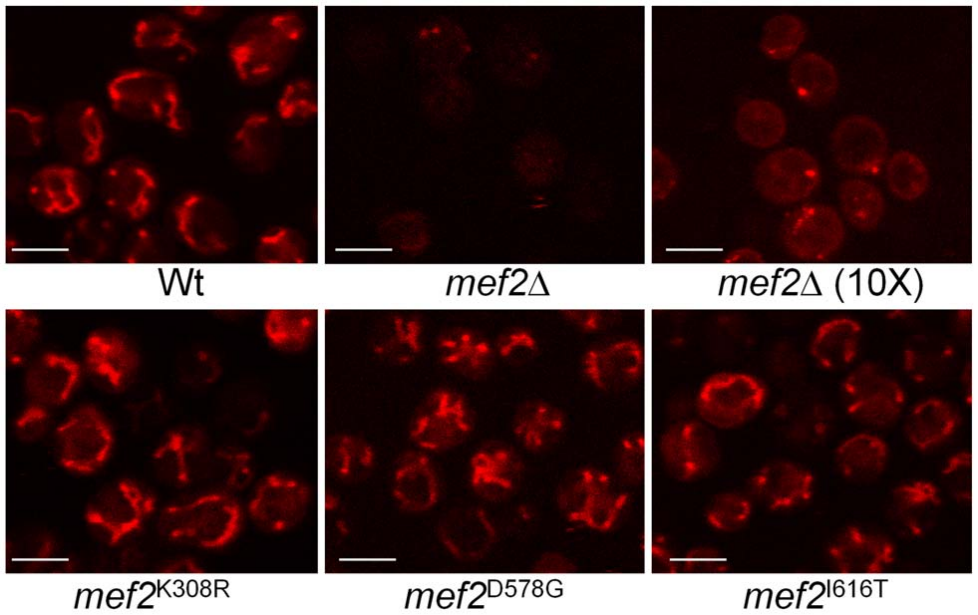
Visualisation of mitochondrial membrane potential. Cells were stained with MitoTracker Red CMXRos and observed using a laser scanning confocal microscope. To better visualise mitochondrial structure within the *mef2* deletant, cells were stained with a 10× concentration of MitoTracker Red. Scale bar represents 5 µm.

Interestingly, the partially respiratory deficient *mef2* mutants exhibit a greater statin sensitivity than the ethidium bromide generated ρ^0^ strains, which are completely devoid of respiratory function (Table 1). This, in support of the *mef2*Δ results, indicates the role of a non-respiratory function of *MEF2* in the statin response. In addition to a lack of mtDNA, we have previously shown, by staining cells with MitoTracker Red CMXRos, that the *mef2*Δ strain has a reduced mitochondrial membrane potential (ΔΨ) and that mitochondria appear fewer, with a tendency to aggregate [30]. The MitoTracker Red CMXRos probe enters the mitochondrial matrix dependent on ΔΨ. This same method was used to test ΔΨ and mitochondrial morphology in the *mef2* mutants. Cells were visualised using a laser scanning confocal microscope and, in contrast to the *mef2*Δ strain, all five *mef2* mutants displayed mitochondria that stain brightly and are arranged in a tubular network, typical of mitochondria in wild-type yeast. Staining of the three partially respiratory deficient mutants is shown in Figure 6. These results indicate that the *mef2* mutations do not disrupt the function of Mef2p in maintaining ΔΨ and so does not explain the enhanced statin sensitivity of these mutants. It is known that statin toxicity can cause loss of ΔΨ [39]. Therefore one possibility is that atorvastatin acts synergistically with a mutated Mef2p to exacerbate loss of ΔΨ and compromise cell viability. However, other mechanisms, such as modulation of the mitochondrial retrograde response, cannot be discounted [40].

### Protein homology modelling of the human EF-G2mt protein predicts function of EF-G2mt protein variants

**Figure 7:**
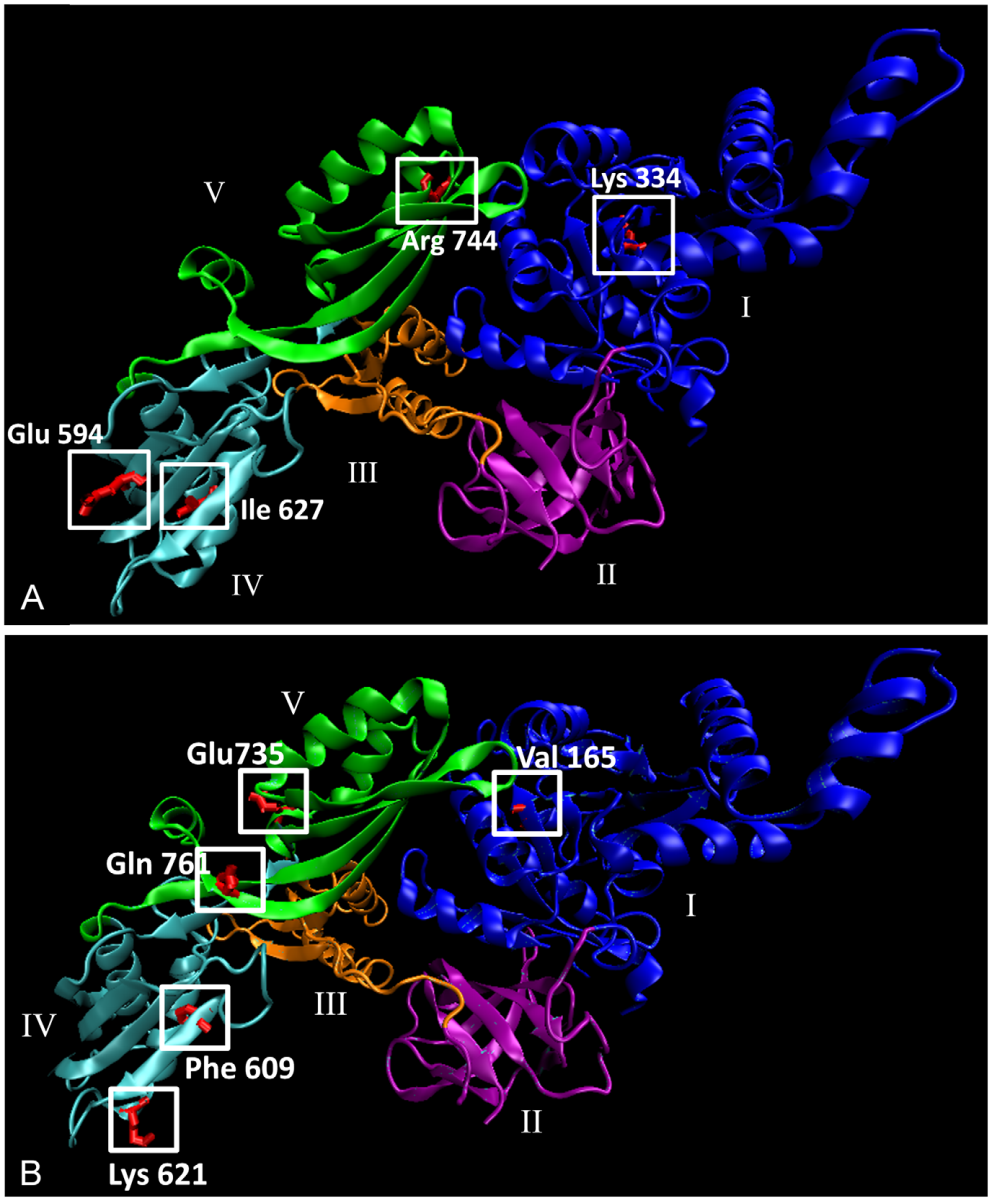
*In silico* model of the human EF-G2mt protein. (A) Model depicting four of the five amino acid variants that were functionally characterised in this study. (B) Five newly discovered variants that have yet to be functionally characterised. Helices are shown as ribbons, beta-sheets are depicted as flat broad arrows and loops and coils appear as thin tubes. The five protein domains of the EF-G2mt protein are distinguished by different colours. Domain I, the GTP binding domain is shown in blue, domain II is purple, domain III is orange, domain IV is cyan and domain V is green. Model was visualised using the Visual Molecular Dynamics program (VMD), version 1.8.7 (University of Illinois).

The crystal structure of the human EF-G2mt protein has not yet been elucidated. Therefore, to gain insight into the molecular effects of the five chosen amino acid variations, a computational model of the EF-G2mt protein was constructed using the SWISS-MODEL server [41]. The model is based on the experimentally determined crystal structure of the *Thermus. thermophilus* EF-G protein [42] which shares 39 percent identity with the human EF-G2mt protein and four of the five EF-G2mt protein domains. As the N-terminal and C-terminal regions of the EF-G2mt protein share particularly low homology with the template, the initial 65 and final 10 amino acid residues could not be accurately modelled. For this reason, the location of amino acid variant *EF-G2mt* ^R774Q^, positioned very close to the C-terminus, was omitted (Figure 7A). Stereochemical quality of the model was assessed by generating a Ramachandran plot using PROCHECK. Eighty seven percent of residues fall within the most favoured regions of the plot, indicating ideal stereochemistry.

Using the *in silico* model, we can make some hypothetical predictions about the function of the protein variants. The human *EF-G2mt* ^K334R^ variant, which corresponds to the respiratory compromised yeast *mef2* ^K308R^ variant, is located on a coil close to the surface of the GTP-binding domain (domain I) that is highly conserved in *EF-G2mt* homologs from all major eukaryotic species. The presence of GTP is essential for ribosome dissociation at the termination of translation, and subsequent hydrolysis of GTP is then required in order to release the EF-G2mt protein from the ribosome [23]. The *EF-G2mt* ^E594G^ and *EF-G2mt* ^I627T^ variants, equivalent to the respiratory compromised *mef2* ^D578G^ and *mef2* ^I616T^ mutants respectively, are both located in domain IV and occur close to the EF-G2mt protein surface. The *EF-G2mt* ^E594G^ variant is expected to diminish the largely negative electrostatic surface potential of this domain, thereby interfering in the protein's interaction with both the mitochondrial ribosome and Rrf1 [23], [25]. The *EF-G2mt* ^I627T^ variant is located on an alpha helix whose structural integrity is disturbed by the threonine hydroxyl group. The final two EF-G2mt protein variants, in which the corresponding *mef2* mutants did not exhibit a phenotype, are located in domain V, the C-terminal region that accelerates (but is not essential for) the ribosome recycling action of the EF-G2mt protein [23].

In the last year, data from the 1000 Genomes project has expanded the number of known polymorphisms in the human genome and a further 11 non-synonymous SNPs have been discovered within the human *EF-G2mt* gene (dbSNP, National Centre for Biotechnology Information (NCBI)) [43]. Of these, one is fully conserved in the *S. cerevisiae* Mef2 protein and another five are semi-conserved (Figure 3 and Figure S2). The location of five of these variants is shown on the EF-G2mt protein model in Figure 7B. Although experimental confirmation is essential, based on the findings above it is hypothesised that the domain IV (*EF-G2mt* ^K621N^ and *EF-G2mt* ^F609Y^) and domain I (*EF-G2mt* ^V165G^) variants will affect protein activity. Therefore, these naturally occurring *EF-G2mt* variants may have respiratory deficient consequences.

## Discussion

By exploiting the genetic tractability of yeast, complemented by siRNA silencing studies in human cell lines, we have identified a mitochondrial translation factor as a mediator of atorvastatin toxicity and also made fundamental discoveries about the function of human variants within the *EF-G2mt* gene. The human *EF-G2mt* gene, ortholog of the yeast *MEF2* gene, was originally identified as a mitochondrial elongation factor gene. However, a recently published comprehensive analysis of the EF-G2mt protein has shown that it functions as a ribosome recycling factor, interacting with the first discovered ribosome recycling factor protein, Rrf1, to dissociate the ribosomal subunits at the termination of translation [23]. In yeast, deletion of the *MEF2* gene results in loss of mtDNA [30], a circumstance which would be lethal in higher eukaryotic cells without the supplementation of uridine and pyruvate [44]. Nevertheless, siRNA silencing experiments show that in contrast to its *RRF1* counterpart, knockdown of human *EF-G2mt* expression does not compromise cell viability in glucose medium. Furthermore, silencing of *EF-G2mt* expression does not deplete cellular mtDNA content. It is possible that some compensatory mechanism enables ribosome recycling to continue to a sufficient degree to maintain cell viability in fermentative cell lines.

When galactose is used as the carbon source instead of glucose, the ATP produced via glycolysis is insufficient for cell energy requirements; therefore, there is a greater reliance on the production of ATP through the oxidative metabolism of glutamine. This more closely resembles the metabolic activity of cells in a human physiological system [37], [45]. By using galactose to force reliance on the mitochondria for cell energy production, it was shown that reduced *EF-G2mt* activity does indeed compromise cell proliferation in respiring cells. It is consequently expected that the *EF-G2mt* gene is essential for cell function in a human system. Furthermore, in support of the notion that abnormalities in mitochondrial function sensitise cells to statin toxicity, the growth defect of *EF-G2mt* silenced cells in galactose medium was even further exacerbated upon exposure to atorvastatin.

For cells in which mitochondrial function is challenged, exposure to mitochondrial toxicants, such as statins, places additional stress on mitochondrial function and this has the potential to trigger pathogenicity. Indeed we have shown that ρ^0^ strains, lacking respiratory capacity, are more sensitive to atorvastatin than those with a functioning mitochondrial genome in both yeast and human cells. Studies have shown that statins exert their mitochondrial toxicity effects by inhibiting function of the electron transport chain but there is also evidence of non-respiratory mitochondrial consequences [46], [47], [48]. These include a loss of mitochondrial membrane potential, aberrant mitochondrial morphology and apoptosis [39]. These effects, in combination with the absence of respiratory function may explain the hypersensitivity of ρ^0^ cells to statin.

The hypersensitivity of respiratory deficient cells to statins may have clinical ramifications for patients that have variations within mitochondrial functioning genes. Statins have been known to aggravate clinically silent disease associated mutations resulting in myopathies. In fact, mutations (which in many cases were asymptomatic) for three common myopathic diseases; carnitine palmitoyltransferase II deficiency, McArdle disease and myoadenylate deaminase deficiency (AMPD deficiency), are thought to be the underlying determinants responsible for statin-induced myopathy in up to 10 percent of patients showing adverse effects [5]. There are also reports of the statin-induced triggering of MELAS syndrome in patients whose MELAS mutations were clinically silent [13], [14]. As MELAS syndrome arises from mutations in mtDNA, it strongly implicates mitochondrial dysfunction in susceptibility to statin toxicity. This is further supported by the identification of two commonly occurring SNPs in the human *COQ2* gene that are associated with an increased risk of statin intolerance [49]. The *COQ2* gene is required for the synthesis of coenzyme Q10, an essential component of the mitochondrial electron transport chain [49].

Despite the presence of a number of SNPs within the *EF-G2mt* gene, the function of these variants for cell fitness had never been investigated. In this study, five *EF-G2mt* equivalent SNPs were selected for functional analysis in the yeast *MEF2* gene. Unlike the *mef2*Δ deletant, all *mef2* mutants grew proficiently on both glucose and the non-fermentable carbon source glycerol. Furthermore, the mutations had no effect on mtDNA stability, ΔΨ or mitochondrial morphology. However, exposure of the mutants to toxic concentrations of atorvastatin has uncovered a phenotype for three of the *mef2* mutants; *mef2* ^K308R^, *mef2* ^D578G^ and *mef2* ^I616T^, equivalent to the *EF-G2mt* ^K334R^, *EF-G2mt* ^E594G^ and *EF-G2mt* ^I627T^ alleles respectively. *In silico* protein homology modelling reveals these three mutations are located in either the GTP binding domain (domain I) of the EF-G2mt protein or an external domain (domain IV) necessary for ribosomal interaction.

Based on the observations that statins exacerbate clinically silent disease associated mutations, it was predicted that the three mutations compromise mitochondrial function in a subtle yet potentially significant way. The subsequent observation that oxygen consumption was significantly reduced in the statin-sensitive *mef2* mutants confirmed this hypothesis and demonstrates a sub-optimal mitochondrial function for the three *EF-G2mt* equivalent *mef2* mutants. This sub-optimal mitochondrial function is expected to contribute to the atorvastatin-sensitive phenotype of the three *mef2* mutants. However, comparison of the statin sensitive phenotype of the *mef2* ^K308R^, *mef2* ^D578G^, *mef2* ^I616T^ and *mef2*Δ mutants with cytoplasmic ρ^0^ strains indicates that statin sensitivity is not fully explained by the reduced respiratory capacity of these mutants and further studies are required to completely elucidate the precise mechanism.

This study constitutes the first report of a phenotype associated with *EF-G2mt*, demonstrating an essential role for aerobic respiration in human cell lines and an importance for cell tolerance to atorvastatin. Atorvastatin constituted the focus of this study and is the highest selling and also one of the more potent of the statins [50]. Although the various statins have been shown to differ in their cellular toxicity effects, all of them have been implicated with mitochondrial dysfunction [48], [50], [51], [52], [53]. It would therefore be expected that a mitochondrial mediator of atorvastatin toxicity may also mediate cell response to the other statins. In support of this, preliminary experiments confirm that the atorvastatin sensitive *mef2* mutants also exhibit sensitivity to lovastatin.

With an estimated 38 million people around the world undertaking statin treatment [9], the identification of novel biomarkers for statin toxicity has the potential to personalise therapy for millions of individuals. To date, only a handful of genes have been associated with statin toxicity [9], but the finding of the *EF-G2mt* gene as a potential pharmacogenetic candidate has strengthened the association between existing mitochondrial dysfunction and statin hypersensitivity. Importantly, the discovery of naturally occurring human polymorphisms within the *EF-G2mt* gene that affect respiratory function indicates that these variants, either alone or in combination with other polymorphisms, have significant pathogenic consequences. This opens avenues for further clinical investigations into a possible association between *EF-G2mt* variants and disease.

## Materials and Methods

### Chemicals

Atorvastatin calcium was purchased from 7 Chemicals (India). Stock solutions were prepared by dissolving atorvastatin in methanol at a concentration of 20 mg/mL and solutions were stored at −20°C. 5-fluoroorotic acid and geneticin (G418) were purchased from Sigma.

### Yeast strains and media

The haploid wild-type *S. cerevisiae* strains used were of the background BY4741 (*MAT* **a** *his3Δ1 leu2Δ0 met15Δ0 ura3Δ0*) and BY4742 (*MAT*α *his3Δ1 leu2Δ0 lys2Δ0 ura3Δ0*). The diploid strain was BY4743 (*MAT* **a**/α *his3*Δ*1/his3*Δ*1 leu2*Δ*0/leu2*Δ*0 LYS2/lys2*Δ*0 MET15/met15*Δ*0 ura3*Δ*0/ura3*Δ*0*) [54]. The *mef2*Δ/*MEF2*, *mef2*Δ and the cytoplasmic ρ^0^ strains were previously described [30]. Prior to experiments, yeast strains were cultured in liquid YEPD (1% yeast extract, 2% peptone, 2% dextrose) medium for 24 hours, subcultured and then grown to exponential phase in Synthetic Complete (SC) medium (0.67% Difco yeast nitrogen base without amino acids, 2% dextrose, 0.79 g L^−1^ amino acid supplement (Sunrise Science Products, Australia)). Cells were incubated at 30°C with shaking.

### Statin viability assay in yeast

Triplicate exponential phase cultures grown in SC medium were diluted to an optical density (OD_595 nm_) of 0.2 and 1.25 mL of this culture was added to 3.75 mL SC medium with the appropriate concentration of atorvastatin. After a maximum of five days growth, cells were diluted and plated onto solid YEPD (YEPD plus 2% agar) medium and viability counts were performed 48 hours later.

### Cell culture

HepG2 cells and RD Cells are from the American Type Culture Collection (ATCC). Cells were cultured at 37°C and 5% CO_2_ in Dulbecco's Modified Eagle's Medium (DMEM) medium (Gibco) containing 4.5 g/L glucose and 10% fetal bovine serum (FBS) (Bovogen Biologicals, Australia). For experiments in which galactose was used as the carbon source, cells were grown in glucose free DMEM with 10% fetal bovine serum and 4.5 g/L galactose. To generate the ρ^0^ cell lines, used as respiratory deficient controls, HepG2 and RD cells were cultured for eight weeks in DMEM medium containing 100 ng/mL ethidium bromide and supplemented with 10% FBS, 50 µg/mL uridine and 100 µg/mL sodium pyruvate [31], [44]. Following ethidium bromide treatment, ρ^0^ cells were maintained in medium supplemented with uridine and sodium pyruvate. Depletion of mtDNA was confirmed using qPCR.

### Cell transfection with siRNA

The ON-TARGETplus SMARTpool, comprising of four siRNAs targeting the *EF-G2MT* transcript (NM_170691) was purchased from Dharmacon (cat. # L-017534-01-0005, Dharmacon, Thermo Fisher Scientific, Lafayette, CO.). The corresponding ON-TARGETplus non-targeting SMARTpool (cat. #D-001810-10-05) and DharmaFECT transfection agents were also purchased from Dharmacon. DharmaFECT agent 2 (0.2 µL/100 µL) and DharmaFECT agent 4 (0.4 µL/100 µL) were used to transfect RD cells and HepG2 cells respectively. All siRNAs were used at a final concentration of 25 nM. To transfect cells, equal volumes of the siRNA and transfection agent were mixed, allowed to incubate for 30 minutes, and 100 µL of the solution was added to 400 µL DMEM medium containing 10% FBS. This solution was added to the attached cells which had been washed in PBS. Gene expression silencing was assessed at 72 hours post-transfection by qPCR. For experiments in which cells were re-transfected, the above procedure was performed again at 72 hours subsequent to the initial transfection.

### Cell proliferation assay

At 24 hours post-transfection, approximately 1×10^4^ cells/well of each culture were seeded into eight wells (one for each day of the eight-day proliferation assay) of a 96-well plate and allowed to attach overnight. Each day, the number of viable cells in one of the eight wells was assessed using the CellTiter-Glo assay (Promega) as described above. Cell medium was changed every two days.

### IC_50_ assays

Approximately 5×10^4^ cells/well were seeded into wells of a 96-well plate. For siRNA transfected cells, seeding occurred at 24 hours post-transfection. Following overnight incubation to enable attachment, media was changed to DMEM containing 10% FBS plus the appropriate atorvastatin concentration. Atorvastatin concentrations ranged from 0 to 128 µM for RD cells and 0 to 1024 µM for HepG2 cells. Cell survival after 48 hours in the presence of atorvastatin was estimated using the CellTiter-Glo luminescent cell viability assay (Promega), which measures intracellular ATP concentration. The assay was performed according to the manufacturer's instructions and luminescence was quantified using a Tecan Genios microplate reader. IC_50_ values were calculated from dose-response curves that were generated using least-squares linear regression.

### Construction of SNP mutations in yeast *MEF2* gene

Codon modifications used to alter yeast Mef2p amino acid residues to those of the corresponding EF-G2mt variants are A2305C, A2219G, T1848C, A1734G and A924G for the yeast *mef2* ^K769Q^, *mef2* ^R740G^, *mef2* ^I616T^, *mef2* ^D578G^ and *mef2* ^K308R^ respectively. The GenBank accession number for the *MEF2* sequence used was NC_001142.9. The single base pair substitutions were created in the yeast *MEF2* gene according to the double-strand break mediated *delitto perfetto* method [55]. The pGSKU plasmid containing the CORE-I-*Sce*I cassette was kindly provided by Francesca Storici (Georgia Tech, Atlanta, GA). The CORE-I-*Sce*I cassette was PCR amplified with chimeric primers (Table S2) that contain 50 bp homologous to the site of insertion and 20 bp for amplification of the cassette. Cassette amplification was performed in 50 µL PCR reactions (25 µL 2× Phusion Flash PCR Master mix (Finnzymes), 0.5 µM each primer and approximately 1–10 ng purified pGSKU plasmid) using a PTC-200 thermal cycler (MJ Research) (Initial denaturation, 98°C for 10 seconds; denature, 98°C for 1 second; anneal/extend, 72°C for 75 seconds; repeat denature and anneal/extend for 30 cycles; final extension, 72°C for 1 min). Cells were transformed with 10 µL of the concentrated PCR product as previously described [55]. Transformants were selected by plating onto synthetic medium lacking uracil (0.67% Difco yeast nitrogen base without amino acids, 2% dextrose, 0.79 g L^−1^ uracil dropout amino acid supplement (Sunrise Science Products, Australia) and 2% agar) and after 24 hours, G418 resistance was checked by replica plating onto solid YEPD containing 200 µg/mL G418 (Sigma). Colony PCR was performed to confirm accurate integration of the cassette. Cells containing the integrated CORE-I-*Sce*I cassette were then transformed with 0.5 nM of each strand of a pair of complementary 80 bp oligonucleotides (Table S2) containing the desired substitution and possessing 40 bp on either side of the oligo which is targeted to the regions adjacent to the integrated marker (GeneWorks, Adelaide). To induce the I-*Sce*I mediated double strand break at the recombination site, prior to transformation cells were cultured in 50 mL of synthetic complete medium containing 2% galactose instead of glucose and incubated at 30°C with shaking for a period of six to eight hours. Following transformation, cells were plated onto solid YEPD medium and after 24 hours, replica plated onto synthetic complete medium containing 1 g/L 5-fluoroorotic acid and 60 mg/L uracil to check for loss of the *URA3* marker. Loss of the CORE-I-*Sce*I cassette was confirmed using colony PCR (primers listed in Table S2) and the resulting PCR product was sequenced to ensure the desired mutation was present. Due to complete disruption of the *MEF2* gene during insertion of the cassette, all resulting *mef2* mutants were lacking mtDNA. Therefore, to reintroduce mtDNA, mutant strains were mated with the wild-type of opposite mating type (the BY4741 strain (*MAT* **a**)). Diploid strains were then sporulated and dissected, resulting in 2∶2 segregation of wild-type and *mef2* mutant colonies, all of which were ρ^+^. Sequencing was used to identify the colonies which possessed the desired mutation.

### Measurements of oxygen utilisation

Cell respiration was measured by monitoring dissolved oxygen levels in 30 mL of exponential phase yeast cells at an optical density of 0.2 in YEPD medium. Cells were cultured in 50 mL Erlenmeyer flasks sealed with a rubber bung to minimise the exchange of oxygen with the external environment. Each flask was equipped with a PreSens PSt3 oxygen sensitive spot (NomaCorc) and the percentage of dissolved oxygen in the medium was measured using an oxoluminescent device, the NOMASense oxygen analyser system (NomaCorc) [35], every 15 minutes until percent oxygen reached below 10% for the wild-type strain.

### Visualisation of mitochondrial membrane potential (ΔΨ)

MitoTracker Red CMXRos (Molecular Probes) was added directly to a 500 µL volume of exponential phase culture in YEPD to a final concentration of 250 nM (1×) or 2500 nM (10×). Cells were incubated at 30°C for 30 minutes, washed with fresh medium and resuspended in YEPD medium. Cells were mounted onto a glass slide and viewed immediately using a laser scanning confocal microscope (Zeiss LSM 710, Carl Zeiss Microimaging, Germany) controlled using the ZEN 2010 software (Carl Zeiss Microimaging, Germany). The excitation line used was 543 nm and the laser power was set at 2%. Cells were viewed using 630× optical magnification and 3× digital magnification. All samples were analysed using the same settings.

### Protein homology modelling

The protein model of the human EF-G2mt protein was based on the crystal structure of the *Thermus thermophilus* EF-G protein (Protein Data Bank 2bm0) [42]. This shares 39% residue identity. Selection of the *T. thermophilus* template and homology modelling was carried out using the SWISS-MODEL server in ‘project mode’ to enable alignment inspection prior to modelling [41]. The completed model was then submitted to the SWISS-MODEL suite of quality check programs which tests for model quality and stereochemistry using algorithms such as ANOLEA [56] and PROCHECK [57]. The model was visualised using the Visual Molecular Dynamics program (VMD), version 1.8.7 (University of Illinois) and this software was also used for the assignment of protein secondary structure and the assessment of electrostatic potential.

### Bioinformatics

All sequences, both yeast and human, were obtained from the Ensembl database. There are three known human EF-G2mt protein isoforms but isoform I (AAH15712.1) was used throughout this study. Human *EF-G2mt* SNPs were identified using the dbSNP database available on the National Center for Biotechnology Information (NCBI) website (www.ncbi.nlm.nih.gov/SNP/index.html). The Lalign program available on the Swiss EMBnet server (www.ch.embnet.org/software/lalign_form.html) was used to generate global alignments of protein sequences, based on the BLOSUM50 matrix [58]. Graphical representations were constructed using the BioEdit version 7.0.5 sequence alignment editor.

## Supporting Information

Figure S1 Quantitative PCR analysis of mtDNA levels for *EF-G2mt* siRNA transfected RD cells relative to the non-targeting siRNA control. The mtDNA/nDNA ratio was determined using qPCR at 72 hours post-transfection. Cells were re-transfected and mtDNA was analysed again following another 72 hours. The nuclear *r18S* gene and the mitochondrial *ND1* gene were used to determine the quantity of nuclear DNA and mtDNA respectively.
(TIF)

Figure S2 Global protein alignment of human EF-G2mt and yeast Mef2p. Red shaded boxes along the EF-G2mt protein sequence indicate amino acid variations resulting from non-synonymous SNPs in the human *EF-G2mt* gene. A corresponding red shaded box in the yeast Mef2p alignment designates a fully conserved amino acid residue and a blue shaded box represents a semi-conserved residue.
(PDF)

Figure S3 Quantitative PCR analysis of mtDNA levels of the yeast *mef2* mutants relative to the wild-type. The mtDNA/nDNA ratio was determined using qPCR. There was no detection of mtDNA in the ρ^0^ *mef2*Δ deletion mutant, denoted ND. Data represents the mean ± SEM (n = 3). The nuclear *ACT1* gene and the mitochondrial *COX1* gene were used to determine the quantity of nuclear DNA and mtDNA respectively.
(TIF)

Table S1 Growth rate of *mef2* mutants in glucose and glycerol medium. Triplicate exponential phase cultures were diluted to an optical density (OD_595 nm_) of 0.2 and 50 µl of this culture added to 150 µl of YEPD in wells of a 96-well plate. Cell growth was monitored by measuring OD at 595 nm every hour for 15 hours in a Tecan Genios microplate reader. Cell doubling time was calculated using the formula ln 2/k, where k is the maximal slope of the curve when ln(OD_595_) is plotted against time. Data represent the mean cell doubling time ± SEM (n = 3). A one-way ANOVA was used to compare mean cell doubling time of mutants with that of the wild-type. ***P<0.001.
(PDF)

Table S2 Oligonucleotides used in this study.
(PDF)
