# Supplementary material for: Polymorphisms in the Mitochondrial Ribosome Recycling Factor EF-G2mt/MEF2 Compromise Cell Respiratory Function and Increase Atorvastatin Toxicity
Source: PLoS Genet. 2012 Jun 14;8(6):e1002755. doi: 10.1371/journal.pgen.1002755 (PMC3375252; doi:10.1371/journal.pgen.1002755)
Supplement: Figure S2 — Global protein alignment of human EF-G2mt and yeast Mef2p. Red shaded boxes along the EF-G2mt protein sequence indicate amino acid variations resulting from non-synonymous SNPs in the human EF-G2mt gene. A corresponding red shaded box in the yeast Mef2p alignment designates a fully conserved amino acid residue and a blue shaded box represents a semi-conserved residue. (PDF) [file pgen.1002755.s002.pdf]

Global alignment generated using Lalign (Huang & Miller 1991).

```
                                        10        20        30
Mef2p --------------------------MWKWNVRRWAGARVNISKNRLSVINVGSRYLSTA
                                  . ...:    .: ...:  :. .. .  ...
EF-G2 MLTNLRIFAMSHQTIPSVYINNICCYKIRASLKRLK-PHVPLGRNCSSLPGLIGNDIKSL
              10        20        30         40        50

              40        50        60        70        80        90
Mef2p RS----PLSKVRNIGIIAHIDAGKTTTTERMLYYAGISKHIGDVDTGDTITDFLEQERSR
      .:    :..:.:::::.:::::::::::::.:::.: .. .:::: :::.:::. ::: :
EF-G2 HSIINPPIAKIRNIGIMAHIDAGKTTTTERILYYSGYTRSLGDVDDGDTVTDFMAQERER
     60        70        80        90       100       110
  p.His60Asp
             100       110       120       130       140       150
Mef2p GITIQSAAISFPWRNTFAINLIDTPGHIDFTFEVIRALKVIDSCVVILDAVAGVEAQTEK
      ::::::::..: :.. . .::::::::.:::.:: : :.:.:. :...:: :::::::
EF-G2 GITIQSAAVTFDWKG-YRVNLIDTPGHVDFTLEVERCLRVLDGAVAVFDASAGVEAQTLT
    120       130        140       150       160       170
                                                p.Val165Gly

               160       170       180       190       200
Mef2p VWKQSKSK--PKICFINKMDRMGASFNHTVNDLINKFMRGTTTKPVLVNIPYYRKQPTSN
      ::.:. ..  :.:::.::::. ::::...:... .:.     .::.:...:  . .
EF-G2 VWRQADKHNIPRICFLNKMDKTGASFKYAVESIREKL----KAKPLLLQLPIGEAK----
     180       190       200       210           220       230

     210       220       230             240       250       260
Mef2p DYVFQGVIDVVNGKRLTWNPENPD------EIIVDELDGTSLEQCNRCRESMIETLTEYD
        .:.::.:::  ..: :: .. :      . ...  :   :.. .. :...:: ... :
EF-G2 --TFKGVVDVVMKEKLLWNCNSNDGKDFERKPLLEMNDPELLKETTEARNALIEQVADLD
               240       250       260       270       280

           270       280       290       300       310       320
Mef2p EDLVQHFLEEAEGDYSKVSAQFLNASIRKLTMKNMIVPVLCGASFKNIGVQPLLDAIVNY
      .....  :::   ... . :. :...:...:. .  ::::::...:: :.::::::.. :
EF-G2 DEFADLVLEEFSENFDLLPAEKLQTAIHRVTLAQTAVPVLCGSALKNKGIQPLLDAVTMY
     290       300       310       320       330       340
                                                p.Lys334Arg

           330       340       350       360       370       380
Mef2p LPSPIEAELPELNDKTVPMKYDPKVGCLVNNNKNLCIALAFKVITDPIRGKQIFIRIYSG
      ::::      : : . .    :           .:: ::::::. :  ::  .:.:::::
EF-G2 LPSP-----EERNYEFLQWYKD-----------DLC-ALAFKVLHDKQRGPLVFMRIYSG
     350            360                   370       380       390

           390       400       410       420       430       440
Mef2p TLNSGNTVYNSTTGEKFKLGKLLIPHAGTSQPVNILTAGQIGLLTGSTVENNISTGDTLI
      :..   ...: . .   ....::.: :     .  ::::.:.: .:    .. .::::..
EF-G2 TIKPQLAIHNINGNCTERISRLLLPFADQHVEIPSLTAGNIALTVGL---KHTATGDTIV
             400       410       420       430          440

                    450       460        470       480       490
Mef2p THSS---------KKDGLKSLDKKKELT-LKINSIFIPPPVFGVSIEPRTLSNKKSMEEA
      . .:         ...: :.  ...:   : . .. :: :::  .::: .::..  ..:.:
EF-G2 SSKSSALAAARRAEREGEKKHRQNNEAERLLLAGVEIPEPVFFCTIEPPSLSKQPDLEHA
      450       460       470       480       490       500
```

```
           500       510       520       530       540       550
Mef2p LNTLITEDPSLSISQNDETGQTVLNGMGELHLEIAKDRLVNDLKADVEFGQLMVSYKETI
      :. :  :::::..  . ..::::: ::::::.:: .::.  .   .. .: :.:.:.:::
EF-G2 LKCLQREDPSLKVRLDPDSGQTVLCGMGELHIEIIHDRIKREYGLETYLGPLQVAYRETI
     510       520       530       540       550       560

            560       570       580       590       600       610
Mef2p -NSETNIETYESDDGYRFSLSLLPNSDALPNCLAYPLGVNENFLIMEKNGNWDKEWKYQV
       ::    .: .   : .  :  . . .: :   .  . : : :   :. .    :   .:
EF-G2 LNSVRATDTLDRTLGDKRHLVTV-EVEARPIETSSVMPVIE-FEYAESIN----EGLLKV
     570       580       590        600        610           620
                            p.Glu594Gly     p.Phe609Tyr    p.Lys621Asp

            620       630       640       650       660       670
Mef2p SFESILNSIIASCIVGLQRGGKIANFPLYACSIKINSDWSVPPDIETPQEILKITRNLIF
      : :.: :.: ..:. :   :.     :.   .: ..:  .. :   : .    ..:  . 
EF-G2 SQEAIENGIHSACLQGPLLGS-----PIQDVAITLHS-LTIHPGTSTTMISACVSRC-VQ
           630       640            650        660       670
       p.Ile627Thr

            680       690       700       710        720       730
Mef2p KALNDLKPEKYNLLEPIMNLDLTIPQSDVGTVLQDLTGAR-KAQILSIEDESSVSNSGAS
      :::.  : .: ..:::.:::..:. .. .. :: ::.  : . : .. ....:  . . 
EF-G2 KALK--KADK-QVLEPLMNLEVTVARDYLSPVLADLAQRRGNIQEIQTRQDNKVVIGFVP
          680        690       700       710       720       730

               740       750        760       770       780
Mef2p TCNSPENSN--RIYIPSDAVTTLH-ATKDKKNTQETSSNVKKIIKAKVPLREITTYTNKL
        .    :.  :    ..:. .:. .: .  : :. .. ...
EF-G2 LAEIMGYSTVLRTLTSGSATFALELSTYQAMNPQDQNTLLNRRSGLT-------------
           740       750       760       770
    p.Glu735Gly p.Arg744Gly    p.Gln761Lys  p.Arg774Gln

       790       800       810
Mef2p RSLSQGRGEFNIEYSDMEKVTNDRLQSILHDL

EF-G2 --------------------------------
```
