# Supplementary material for: Polymorphisms in the Mitochondrial Ribosome Recycling Factor EF-G2mt/MEF2 Compromise Cell Respiratory Function and Increase Atorvastatin Toxicity
Source: PLoS Genet. 2012 Jun 14;8(6):e1002755. doi: 10.1371/journal.pgen.1002755 (PMC3375252; doi:10.1371/journal.pgen.1002755)
Supplement: Table S1 — Growth rate of mef2 mutants in glucose and glycerol medium. Triplicate exponential phase cultures were diluted to an optical density (OD595 nm) of 0.2 and 50 µl of this culture added to 150 µl of YEPD in wells of a 96-well plate. Cell growth was monitored by measuring OD at 595 nm every hour for 15 hours in a Tecan Genios microplate reader. Cell doubling time was calculated using the formula ln 2/k, where k is the maximal slope of the curve when ln(OD595) is plotted against time. Data represent the mean cell doubling time ± SEM (n = 3). A one-way ANOVA was used to compare mean cell doubling time of mutants with that of the wild-type. ***P<0.001. (PDF) [file pgen.1002755.s004.pdf]

Table S1: **Cell doubling time of *mef2* mutants grown on fermentable and non-fermentable carbon sources**

	**Wild-type**	***mef2*Δ**	**K769Q**	**R740G**	**I616T**	**D579G**	**K308R**
**Glucose**	2.90 ± 0.07	3.97 ± 0.09***	2.87 ± 0.12	2.87 ± 0.12	2.85 ± 0.12	2.83 ± 0.18	2.84 ± 0.17
**Glycerol**	12.6 ± 0.21	Growth absent	12.5 ± 0.16	12.2 ± 0.13	12.1 ± 0.27	12.2 ± 0.15	12.4 ± 0.23
