# Supplementary material for: Polymorphisms in the Mitochondrial Ribosome Recycling Factor EF-G2mt/MEF2 Compromise Cell Respiratory Function and Increase Atorvastatin Toxicity
Source: PLoS Genet. 2012 Jun 14;8(6):e1002755. doi: 10.1371/journal.pgen.1002755 (PMC3375252; doi:10.1371/journal.pgen.1002755)
Supplement: Table S2 — Oligonucleotides used in this study. (PDF) [file pgen.1002755.s005.pdf]

Table S2: Oligonucleotides used in this study

Primer name	Primer sequence 5'----3'
**PCR primers used to amplify the CORE-I-*Sce*I cassette** (blue denotes homology to *MEF2* site of insertion, green indicates I-*sce*I restriction site)	
pGSKU K769Q F	ACGCAACCAAAGATAAAAAAAACACTCAAGAGACCAGTTCT AATGTAAAATTCGTACGCTGCAGGTCGAC
pGSKU K769Q R	TTGGTGTAGGTGGTAATTTCCCTTAGTGGCACTTTCGCTTTTA TAATTTTTAGGGATAACAGGGTAATCCGCGCGTTGGCCGATT CAT
pGSKU R740G F	CGTGTCAAATTCTGGCGCATCCACTTGTAATTCTCCAGAGAA CAGCAATATTCGTACGCTGCAGGTCGAC
pGSKU R740G R	ATCTTTGGTTGCGTGTAGGGTTGTAACAGCATCAGAGGGAAT ATATATCCTAGGGATAACAGGGTAATCCGCGCGTTGGCCGA TTCAT
pGSKU I616T F	AATGGTAATTGGGATAAAGAATGGAAATACCAAGTTTCTTT CGAATCAATTTCGTACGCTGCAGGTCGAC
pGSKU I616T R	TTCCGCCTCTTTGAAGTCCCACAATGCAACTTGCAATAATAG AATTTAGATAGGGATAACAGGGTAATCCGCGCGTTGGCCGA TTCAT
pGSKU D578G F	GAGAGTGATGATGGATATAGATTCTCCTTATCTCTGCTACCA AATTCCGATTCGTACGCTGCAGGTCGAC
pGSKU D578G R	GAAAATTCTCGTTGACTCCTAATGGATATGCAAGGCAGTTAG GAAGGGCATAGGGATAACAGGGTAATCCGCGCGTTGGCCGA TTCAT
pGSKU K308R F	CTGACTATGAAGAATATGATTGTACCTGTCTTATGCGGTGCG TCTTTTAATCGTACGCTGCAGGTCGAC
pGSKU K308R R	AAGGGAGATAATTAACTATAGCATCTAATAGAGGCTGCACG CCAATATTTTAGGGATAACAGGGTAATCCGCGCGTTGGCCG ATTCAT
**80bp Integrative recombinant oligos containing desired mutation** (point mutation highlighted in red)	
K769Q IRO 1	GATAAAAAAAACACTCAAGAGACCAGTTCTAATGTAAAACAAA TTATAAAAGCGAAAGTGCCACTAAGGGAAATTACCAC
K769Q IRO 2	GTGGTAATTTCCCTTAGTGGCACTTTCGCTTTTATAATTTGTTTTA CATTAGAACTGGTCTCTTGAGTGTTTTTTTTATC
R740G IRO 1	TCTGGCGCATCCACTTGTAATTCTCCAGAGAACAGCAATGGGAT ATATATTCCCTCTGATGCTGTTACAACCCTACACGC

R740G IRO 2	GCGTGTAGGGTTGTAACAGCATCAGAGGGAATATATATCCCATT GCTGTTCTCTGGAGAATTACAAGTGGATGCGCCAGA
I616T IRO 1	GGGATAAAGAATGGAAATACCAAGTTTCTTTCGAATCAACTCTA AATTCTATTATTGCAAGTTGCATTGTGGGACTTCAA
I616T IRO 2	TTGAAGTCCCACAATGCAACTTGCAATAATAGAATTTAGAGTTG ATTCGAAAGAAACTTGGTATTTCCATTCTTTATCCC
D578G IRO 1	GATGGATATAGATTCTCCTTATCTCTGCTACCAAATTCCGGTGCC CTTCCTAACTGCCTTGCATATCCATTAGGAGTCAA
D578G IRO 2	TTGACTCCTAATGGATATGCAAGGCAGTTAGGAAGGGCACCGGA ATTTGGTAGCAGAGATAAGGAGAATCTATATCCATC
K308R IRO 1	AGAATATGATTGTACCTGTCTTATGCGGTGCGTCTTTTAGAAATA TTGGCGTGCAGCCTCTATTAGATGCTATAGTTAAT
K308R IRO 2	ATTAACTATAGCATCTAATAGAGGCTGCACGCCAATATTTCTAA AAGACGCACCGCATAAGACAGGTACAATCATATTCT

PCR confirmation of cassette integration and sequencing confirmation of mutation

K769Q conf F	GACGAATCAAGCGTGTCAAA
K769Q conf R	GGCCACGAGGAACAAGAATA
R740G conf F	GCTCTCAATGACTTAAAACCTGAA
R740G conf R	CTCCCTTGCGATAAGCTCCT
I616T conf F	CTTGAAATTGCCAAGGATCG
I616T conf R	TGAGCCTTTCTTGCTCCTGT
D578G conf F	TCGATGGAAGAAGCTTTAAACA
D578G conf R	TGACCAATCGCTATTGATTTTG
K308R conf F	TTTTCCAAGGTGTTATTGACG
K308R conf R	TACCCCTGATTGGATCCGTA
MAT locus	AGTCACATCAAGATCGTTTATGG

Q-PCR mtDNA copy number analysis (yeast)

nACT1 F	GTATGTGTAAAGCCGGTTTTG
nACT1 R	CATGATACCTTGGTGTCTTGG
mtCOX1 F	CTACAGATACAGCATTTCCAAGA
mtCOX1 R	GTGCCTGAATAGATGATAATGGT

Q-PCR Determination of *EFG2* silencing

EFG2 F	CCACTGGTTTTTATGCGCATT
EFG2 R	CTCTCCGTGCAGTTTCCATTAA
18S F	CCGCTAGAGGTGAAATTCTTG
18S R	CATTCTTGGCAAATGCTTTCG
**Q-PCR mtDNA copy number analysis (human)**	
mtND1 Forward	CCCTAAAACCCGCCACATCT
mtND1 Reverse	GAGCGATGGTGAGAGCTAAGGT
